# Cervical spine alignment following surgery for adolescent idiopathic scoliosis (AIS): a pre-to-post analysis of 81 patients

**DOI:** 10.1186/s12893-019-0471-2

**Published:** 2019-01-15

**Authors:** W. Pepke, H. Almansour, R. Lafage, B. G. Diebo, B. Wiedenhöfer, F. Schwab, V. Lafage, M. Akbar

**Affiliations:** 1 Clinic for Orthopaedics and Trauma Surgery, Center for Orthopaedics, Trauma Surgery and Spinal Cord Injury, Heidelberg University Hospital, Schlierbacher Landstr.200a, 69118 Heidelberg, Germany; 2 Hospital for Special Surgery, New York, NY USA; 3 Department of Orthopaedic Surgery, State University of New York, Downstate Medical Center, Brooklyn, NY USA; 4 Spine Surgery, ATOS Clinic Heidelberg, Bismarckstr. 9-15, 69115 Heidelberg, Germany

**Keywords:** Cervical alignment, Cervical spine, Adolescent scoliosis, AIS, Deformity

## Abstract

**Background:**

Several studies have emphasized the importance of restoring thoracic kyphosis (TK) in the setting of AIS, but very few have discussed changes in cervical spine alignment following surgery. Aim of this study was to evaluate reciprocal cervical alignment change after modification of global and regional thoracolumbar alignment with surgery in the setting of adolescent idiopathic scoliosis (AIS).

**Methods:**

Baseline and 2-yrs follow-up radiographs of AIS patients (*n* = 81) were analysed measuring cervical parameters (upper cervical: C2-C0, McGregor Slope; lower cervical: C2-C7, C2-C7 sagittal vertical axis (SVA), C2-T3, C2-T3SVA, C2-T1Harrison (C2-T1Ha), T1 Slope (T1S)), thoracic, lumbar, pelvic and global alignment parameters. Post-operatively, patients were grouped twice; based on changes in TK and SVA. Cervical alignment was compared between groups. Pearson correlation was conducted to examine the relationship between changes in TK, SVA, and cervical alignment.

**Results:**

Stratification by change in TK, revealed significant alteration of lower cervical alignment T1S [*p* < 0.001]), C2-T3 [*p* = 0.019], C2-T1Ha [*p* = 0.043]), but there was no reciprocal change in the upper cervical spine. Stratification by SVA revealed a significant coexisting change in the lower cervical spine (T1S [*p* < 0.001], C2-C7SVA [*p* = 0.034], C2-T3 [*p* = 0.023], C2-T3SVA [*p* = 0.001]). SVA change was not associated to a change in the upper cervical spine. The correlation analysis showed that with a post-operative increase in TK, the cervical spine became more lordotic. Changes in TK were significantly correlated with: ΔT1S, ΔC2-C7, ΔC2-T3, and ΔC2-T3SVA. Similarly, increased cervical kyphosis was found when SVA was decreased post-operatively. Furthermore, there was a significant correlation between change of SVA and both ΔC2-T3 and ΔC2-T3SVA.

**Conclusions:**

In surgically treated AIS patients, changes in global and regional alignment of the thoracolumbar and cervical spinal segments exhibit interdependence**.** Thus, surgical planning with regard to sagittal deformity in AIS patients should account for the post-operative impact on cervical alignment.

## Background

Spine research is in the midst of a paradigm shift focused on the physiological curvature of the cervical spine [1, 2]. A lordotic cervical curvature is no longer considered to be the sole model of normal physiology. Recent studies support the notion that a kyphotic cervical spine can also represent normal alignment [3, 4]. This controversy is pervasive in studies of both the general population [5, 6] and scoliosis patients [7, 8]. The lack of consensus on what constitutes normal cervical spine alignment may be explained by an incomplete understanding of how regional, global and cervical alignments interact.

Another layer of complexity is added in the setting of adolescent idiopathic scoliosis (AIS), a three-dimensional (3D) deformity that alters thoracolumbar spinal alignment with incompletely studied effects on cervical alignment [7–10]. Thus, at present it is difficult to distinguish physiological from pathological cervical spine alignments in AIS patients. The known global pathologic effects of scoliosis include a deforming coronal deviation of the spine with a concomitant rotation of vertebrae and a flattening of the sagittal profile [11, 12]. In recent studies of adult patients, it has been shown that sagittal alignment has a strong correlation with health-related quality of life scores [13]. Consequently, surgical planning for AIS patients has adopted a focus on sagittal alignment as an important parameter from adult spinal deformity research. In current practice, the use of rod pre-contouring (Cotrel-Dubousset technique) [14], a standard surgical procedure for AIS, provides satisfactory results in the correction of coronal deformities [15–17], but often fails to correct sagittal deformities or restore “normal” thoracic kyphosis (TK).

The relationship between cervical and thoracic sagittal alignment was initially proposed by Hilibrand et al. in 1995, showing a significant correlation between the loss of TK and the development of cervical kyphosis for the entire study group [18]. Recent studies have identified a similar correlation in AIS patients [7, 19–21]. Others have associated deterioration in cervical alignment, including loss of lordosis or development of cervical kyphosis, to the development of axial neck pain and disability [22].

We hypothesized that post-operative changes in cervical alignment correlate with changes in lumbar, thoracic, and global alignment. The purpose of this study was to investigate reciprocal changes in cervical alignment following AIS surgery and whether those changes correlate with the thoracolumbar profile.

## Methods

### Patient population

**Fig. 1:**
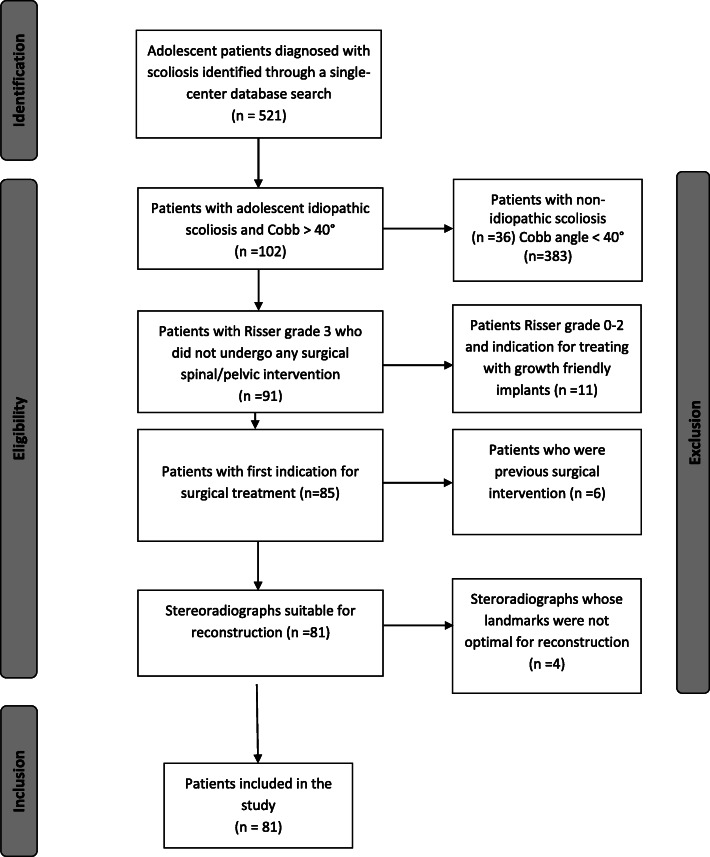
Flow diagram illustrating the process of inclusion and exclusion of study cohort

This is a retrospective single-center study of AIS patients who were treated surgically between 2008 and 2014. Patients underwent anterior-posterior (AP) and lateral full-length x-rays of the spine at baseline and 2-year follow up. X-rays were taken in the standing position, with patients barefoot and holding their upper extremities crossed over their chests. In order to reduce any inaccuracies due to head motion during acquisition of the radiographs, patients were instructed to look straight ahead in a relaxed position. All x-rays that did not fulfill these requirements were excluded. Further exclusion criteria were diagnoses of neuropathic or congenital scoliosis (Fig. 1).

All data was saved as a DICOM (Digital Imaging and Communications in Medicine) file and exported from PACS (Picture Archiving and Communication System) to validated software to be analysed. Assessment of spino-pelvic global alignment was performed by a single observer using SpineView® software [23].

The ethics committee of the medical faculty of Heidelberg University approved this study. Vote no. S-378/2016. Radiographs of our study cohort were conducted routinely. i.e. no additional radiographs were performed in the context of this study. These radiographs were retrospectively analysed. Hence, no informed consent of the participants was required to perform this study.

### Surgical technique

Curve correction was performed in all patients with pedicle screw constructs. All surgeries were performed by two senior authors using a posterior midline incision with subperiosteal dissection. All screws were placed using the freehand technique [24] based on specific anatomical landmarks. The restoration of the coronal and sagittal curves was performed following Cotrel-Dubousset technique [14] using a cobalt chrome rod for the concave side and standard titanium rod for the convex side. All surgeries were performed under neurological monitoring using a triggered electromyogram device. All subjects remained free from any post-operative neurological impairment.

### Data collection and radiographic analysis

**Fig. 2:**
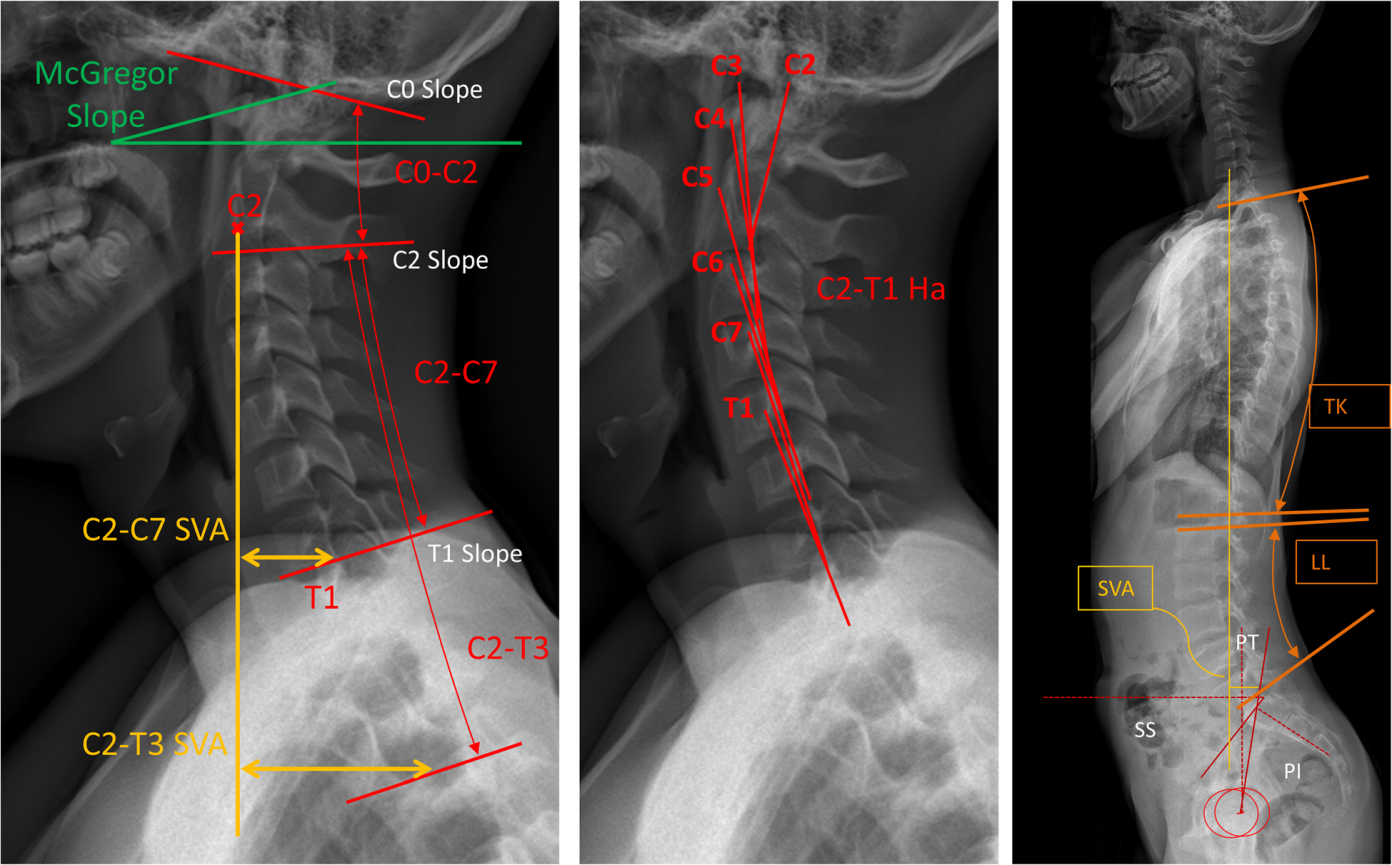
Lateral radiographs illustrating cervical spine parameters. C2-T1 Ha = C2-T1 Harrison posterior tangent method. PT = pelvic tilt; PI = pelvic incidence; SS = sacral slope; LL = lumbar lordosis; TK = thoracic kyphosis; SVA = sagittal vertical axis; MGS = McGregor slope

Demographic and clinical characteristics of patients were obtained from medical records. Radiographic parameters included: spino-pelvic parameters [25] (pelvic incidence [PI], pelvic tilt [PT], sacral slope [SS], sagittal vertical axis [SVA], T1 spino-pelvic inclination [T1SPi], T1 pelvic angle [TPA]); regional alignment (lumbar lordosis [LL], thoracic kyphosis [TK]: T2 to T12); lower cervical (C2-C7, C2-C7 SVA, C2-T3, C2-T3 SVA, C2-T1 Harrison measurement [C2-T1-Ha] [26]) (Fig. 2); and upper cervical alignment parameters (C0 Slope, C2 Slope, C2-C0 Cobb angle, McGregor Slope [MGSlope] [27]) (Table 1). Negative values for angles denote kyphosis.

**Table 1:** Description of the measured parameters with their respective normative values and range

Parameter	Description and clinical relevance	Normative values	range
SS [41]	The angle between the sacral endplate (S1) and the horizontal. In asymptomatic subjects, SS correlates with PI (*r* = 0.81).	41° ±8°	–
PT [41]	The angle between a line form the center of the femoral head axis to the midpoint of the sacral plate and the vertical line. PT reveals a compensatory mechanism and increases with loss of LL. PT correlates with patient reported outcomes (disability).	13° ±6°	−4.7 to 27°
PI [25]	The angle between a line from the center of the femoral head axis to the midpoint of the sacral plate and the perpendicular line to the sacral plate. PI is a morphological parameter, fixed in adults, and determines spine-pelvic alignment. PI=PT + SS	51.9° ± 10°	35° to 85°
PI-LL [42]	the difference between PI an LL. Estimates the “lack of lordosis” and correlates with disability patient reported outcomes HQRoL	−8° ± °9°	−25° to 15°
LL (L1-S1) [43]	The angle between the upper endplate of L1 and sacral endplate.	60.9° ± 12°	31° to 88°
TK (T2-T12) [41]	The angle between the caudal endplate of T12 and the cranial endplate of T2.	−40.6° ± 10°	0° to − 69°
SVA [44]	The horizontal offset from a plumbline dropped from C7 to the postero-superior corner of S1. SVA strongly correlates to clinical outcomes.	−0.5 ± 25 mm	-60 mm to + 65 mm
T1SPI [45]	The angle between vertical and a line from the center of the femoral head axis to the center of the T1 vertebral body. T1SPI assesses the global spino-pelvic alignment and correlates with clinical outcomes.	−1.35° ± 2.7°	−9.16° to 7.2°
TPA [46]	The angle formed by the center of the sacral endplate, the center of the femoral head axis, and the center of T1 vertebral body. TPA strongly correlates with HRQOL, PT and SVA.	7.7° ± 7°	−6° to 23°
T1 Slope [47]	The angle between the horizontal line and cranial endplate of T1. T1 Slope defines the orientation of the cervical spine and highly correlates with SVA and TK.	26° ± 10°	14° to 38°
CL (C2-C7) [3]	The angle between the caudal endplate of C2 and the caudal endplate of C7. CL increases with positive sagittal malalignment in order to maintain horizontal gaze.	5° ± 12°	−10° to 22°
cSVA (C2-C7 SVA) [2]	The horizontal offset from the plumbline dropped from C2 to the postero-superior corner of C7. cSVA is a descriptor of cervical sagittal deformity.	26 ± 11 mm	12 mm to 40 mm
C2-T3	The angle between the caudal endplate of C2 and the caudal endplate of T3.	–	–
C2-T3 SVA	The horizontal offset from the plumb line dropped from C2 to the postero-superior corner of T3	–	–
C2-T1 – H [22]	Involves drawing lines that are parallel to the posterior surfaces of all cervical vertebral bodies from C2 to T1 and then summing the segmental angles for an overall cervical curvature angle. The Harrison method is a precise method of quantifying cervical curvature.	about 41.8°	–
C2 Slope	The angle between the horizontal line and cranial endplate of C2. C2 Slope defines the orientation of upper cervical spine.	–	–
C0 Slope	The angle between the horizontal and cranial ring of C0. C0 Slope defines the orientation of upper cervical spine.	–	–
MGSlope [48]	The angle between the horizontal line and a line connecting the posterior aspect of the hard palate and the opisthion.	1.8° ± 6.3°	–

*SS* sacral slope, *PT* pelvic tilt, *PI* Pelvic incidence, *LL (L1 – S1)* lumbar lordosis measured from L1 to S1, *PI-LL* pelvic incidence minus lumbar lordosis mismatch, *TK (TK 2 – TK 12)* thoracic kyphosis measured from TK 2 to TK 12, *SVA* sagittal vertical axis, *T1SPi* T1 spinopelvic inclination, *TPA* T1 pelvic angle; cranial, *C0S* C0 Slope, *C2S* C2 Slope, C2-C0 angle; C2-C7 angle, C2-C7 SVA (*SVA* sagittal vertical axis), C2-T3angle, C2-T3 SVA (*SVA* sagittal vertical axis), T1 Slope, *MGS* McGregor Slope (line), *HRQOL* Health-Related Quality of Life, *−--* not estimated

### Patient stratification

Sagittal alignment was compared between baseline and 2-year follow-up. Patients were grouped based on changes in their TK and SVA into: increased TK (ΔTK < − 5°; *n* = 40), stable TK (ΔTK between − 5° to 5°; *n* = 31), and decreased TK (ΔTK > 5°; *n* = 10); increased SVA (ΔSVA> 25 mm; n = 31), stable/neutral SVA (ΔSVA = − 25 mm to 25 mm; *n* = 23); and decreased SVA (ΔSVA<− 25 mm; *n* = 27) [28] (Table 2).

**Table 2:** Stratification based on the change on lumbar lordosis (LL), thoracic kyphosis (TK) and sagittal vertical axis (SVA)

Stratification based on the change in LL, TK and SVA		
Thoracic Kyphosis (TK)		
Increase: ΔTK < −5°	Stable: ΔTK = [− 5°; 5°]	Decrease: ΔTK > 5°
Sagittal Vertical Axis (SVA)		
Increase: ΔSVA> 25 mm	Stable: ΔSVA = [− 25 mm; 25 mm]	Decrease: ΔSVA<− 25 mm

*TK* thoracic kyphosis, *SVA* sagittal vertical axis, *Δ* = parameter change pre- to-post-operatively

### Statistical analysis

Cervical alignment was compared between TK and SVA groups using ANOVA. Pearson correlation analysis was utilized to investigate the relationship between changes in regional/global thoracolumbar alignment and changes in regional cervical alignment. Descriptive statistics were reported as means and standard deviations of the means. The threshold of statistical significance was set at *p* < 0.05. Statistical software package SPSS 20.00 (IBM Corp., Armonk, NY, USA) was used for statistical analysis.

## Results

A total of 81 patients with a mean age of 15.47 (±3.5) years, and 74% female were included in the study. Baseline, postoperative, and Δ (amount of change) of pelvic, thoracolumbar, and cervical sagittal parameters were reported (see Table 3 and Table 4).

**Table 3:** Descriptive analysis of spino-pelvic and global alignment parameters in AIS patients pre- and post-operatively

	Pre-OP				Post-OP				Change = Δ			
Parameter	Min	Max	Mean	SD	Min	Max	Mean	SD	Min	Max	Mean	SD
SS	6.1	62.2	40.5	10.5	12.0	72.6	40.9	11.2	−12.5	17.9	0.4	6.8
PT	−6.0	30.5	12.2	8.1	−17.7	30.5	11.7	9.0	−16.4	10.9	−0.5	6.5
PI	15.2	82.9	52.6	13.7	16.0	81.8	52.5	13.4	−4.8	4.3	−0.1	1.9
PI-LL	−41.2	29.8	−4.9	12.8	−34.5	27.2	−4.3	13.2	−21.5	25.3	0.6	10.6
LL (L1-S1)	20.0	91.7	57.5	12.9	21.7	87.2	56.8	13.6	− 25.9	22.8	−0.7	10.4
TK (T2-T12)	−91.2	17.5	−36.7	17.0	− 93.6	−5.2	−33.1	13.8	−30.9	49.8	−1.1	14.3
SVA	− 111.6	143.5	1.2	46.4	−98.6	168.2	−0.6	45.5	− 140.4	172.9	−1.8	49.3
T1SPI	−11.7	7.4	−4.2	4.3	−15.4	6.5	−4.3	4.0	−15.1	12.5	−0.1	4.6
TPA	−9.3	28.3	8.0	7.4	−16.9	27.2	7.4	8.8	−15.5	14.3	−0.6	6.2

*SS* sacral slope, *PT* pelvic tilt, *PI* Pelvic incidence, *LL (L1 – S1)* lumbar lordosis measured from L1 to S1, *PI-LL* pelvic incidence minus lumbar lordosis mismatch, *TK (TK 2 – TK 12)* thoracic kyphosis measured from TK 2 to TK 12, *SVA* sagittal vertical axis, *T1SPi* T1 spinopelvic inclination, *TPA* T1 pelvic angle; cranial, *MGS* McGregor Slope (line), *Δ* parameter change pre- to post-operatively, *SD* standard deviation

**Table 4:** Descriptive analysis of cervical alignment parameters in AIS patients pre- and post-operatively

	Pre-OP				Post-OP				Change = Δ			
Parameter	Min	Max	Mean	SD	Min	Max	Mean	SD	Min	Max	Mean	SD
T1Slope	81	−8.0	54.3	20.6	81	− 9.1	60.9	23.4	81	−18.6	21.9	2.8
C2-C7	75	−35.2	55.2	−1.7	75	−36.3	59.6	2.8	71	−23.8	53.6	5.5
C2-C7 SVA	75	−5.3	71.6	32.0	75	− 5.1	80.1	34.0	71	−35.6	39.5	2.4
C2-T3	75	−44.0	73.4	−5.3	75	−42.8	68.9	− 4.5	71	− 46.4	42.2	2.0
C2-T3 SVA	75	5.6	123.9	52.7	75	−2.7	165.6	59.2	71	−41.8	48.0	6.8
C2-T1 - H	72	−39.2	79.0	7.3	73	−34.7	78.6	12.4	66	−39.5	49.0	6.1
C2Slope	75	−14.0	47.5	21.9	75	−10.6	53.6	20.6	71	−42.7	34.0	−1.9
C0Slope	42	−16.9	15.9	−0.6	44	−30.8	20.3	−2.3	26	−25.9	8.25	−4.48
C2-C0	40	−6.2	35.4	22.4	44	−9.4	38.6	21.3	26	−20.3	8.63	−2.25
MGSlope	36	−9.3	18.6	4.278	41	−26.3	28.7	3.3	23	−25.8	7.46	−5.05

*C0S* C0 Slope, *C2S* C2Slope, C2-C0angle; C2-C7 angle, C2-C7 SVA (*SVA* sagittal vertical axis), C2-T3 angle, C2-T3 SVA (*SVA* sagittal vertical axis), T1 Slope, *MGS* McGregor Slope (line), *Δ* parameter change pre- to post-operatively, *SD* standard deviation

### Stratification by change in TK (increased by > 5°, changed by < 5°, decreased by> 5°)

**Fig. 3:**
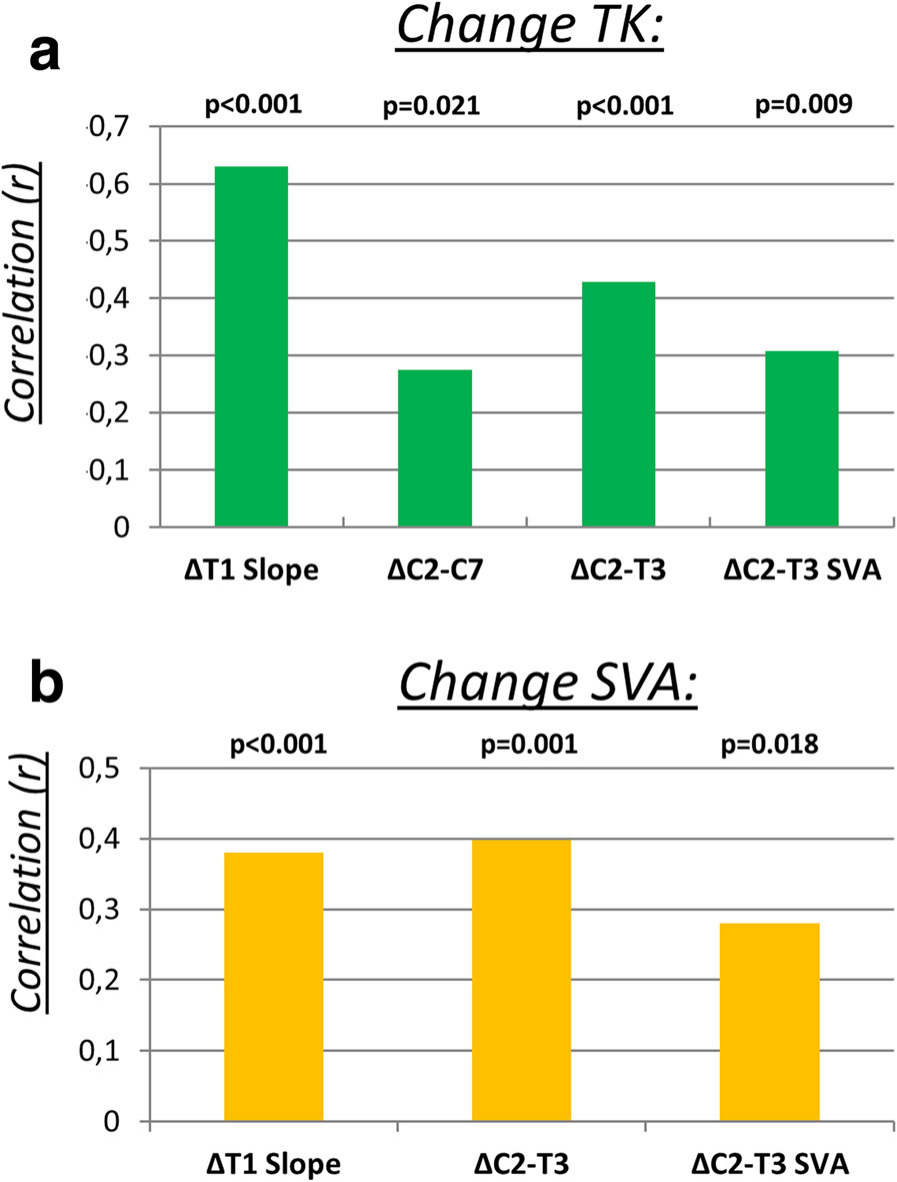
**a** Comparison of subgroups: decreased, stable, and increased thoracic kyphosis (TK) with ΔT1 Slope and ΔC2-T3; *p* = statistical significance, *p* < 0.05. **b** Comparison of subgroups: decreased, stable, and increased sagittal vertical axis (SVA) with ΔT1 Slope, ΔC2-T3, ΔC2-C7 SVA and ΔC2-T3 SVA; *p* = statistical significance, *p* < 0.05

AIS patients whose TK increased post-operatively had a smaller baseline TK (TK: -28° vs. -43° vs. -48°, *p* < 0.001) and a kyphotic cervical spine (C2-T3: -10° vs. -5° vs. 7°, *p* = 0.053). Patients with increased TK post-operatively had more posterior global alignment after surgery (SVA: − 4 mm vs. -7 mm vs. 34 mm, *p* = 0.034; T1SPi: − 5° vs. -5° vs. -1°, *p* = 0.004). Stratifying patients by their pre-to-post-operative TK change (ΔTK) revealed additional associations. Patients with an increase in TK had an increased ΔLL postoperatively (ΔLL: 3° vs. -3.5° vs. -7°, *p* = 0.008), an increase of ΔT1 Slope (ΔT1 Slope: 6° vs. 1° vs. -5°, *p* < 0.001). The same group of patients also exhibited increased lower cervical spine lordosis postoperatively (ΔC2-T3: 6.5° vs.-1° vs. -8°, *p* = 0.019) (Fig. 3a) (see Table 5).

**Table 5:** Stratification by change in thoracic kyphosis (TK): comparison of baseline and postoperative spino-pelvic parameters and their respective pre-to post-op change

	Stratification by change in TK											
	Baseline				Post-OP				Change (Δ)			
	Decrease	Stable	Increase	*p*	Decrease	Stable	Increase	*p*	Decrease	Stable	Increase	*p*
SS	40.7 ± 12.7	39.6 ± 11.3	41.1 ± 9.5	0.835	45.9 ± 13.1	38.9 ± 12.1	41.1 ± 9.9	0.231	5.1 ± 5.2	−0.6 ± 6.5	0.1 ± 6.9	0.054
PT	12.4 ± 11.8	12.5 ± 7.2	11.8 ± 8.0	0.930	8.2 ± 12.8	13.3 ± 7.6	11.2 ± 9.0	0.272	−4.2 ± 5.6	0.8 ± 6.5	−0.6 ± 6.5	0.103
PI	53.2 ± 18.2	52.1 ± 14.5	52.9 ± 12.2	0.964	54.1 ± 17.5	52.2 ± 13.9	52.4 ± 12.2	0.929	0.9 ± 1.8	0.1 ± 1.4	−0.5 ± 2.2	0.072
PI-LL	−9.4 ± 14.3	−7.6 ± 11.1	−1.6 ± 13.2	0.071	−2.6 ± 17.7	−4.0 ± 12.5	−5.0 ± 12.7	0.867	6.8 ± 9.5	3.6 ± 11.2	−3.4 ± 9.0	0.002
LL	62.6 ± 14.6	59.7 ± 12.5	54.5 ± 12.4	0.098	56.7 ± 16.2	56.2 ± 13.2	57.3 ± 13.5	0.945	−5.9 ± 8.8	−3.5 ± 11.2	2.8 ± 9.1	0.008
TK	−48.5 ± 11.8	−43.6 ± 14.0	−28.5 ± 16.4	0.000	−30.7 ± 8.8	−41.5 ± 13.2	−41.0 ± 16.5	0.104	17.9 ± 7.0	2.0 ± 4.4	−12.5 ± 5.3	0.000
SVA	21.4 ± 44.6	−1.4 ± 47.5	−1.8 ± 45.8	0.342	34.0 ± 60.5	−7.2 ± 37.5	−4.0 ± 44.4	0.034	12.6 ± 73.3	−5.8 ± 51.8	−2.2 ± 40.1	0.594
T1SPI	−2.7 ± 5.7	−4.6 ± 4.5	−4.2 ± 3.7	0.463	−0.6 ± 4.5	−5.3 ± 3.4	−4.5 ± 3.9	0.004	2.1 ± 6.1	−0.6 ± 5.0	−0.3 ± 3.8	0.253
TPA	9.7 ± 7.9	8.0 ± 6.0	7.6 ± 8.4	0.737	7.6 ± 12.6	8.1 ± 7.2	6.8 ± 9.0	0.818	−2.1 ± 6.9	0.2 ± 6.1	−0.9 ± 6.1	0.572
T1Slope	25.2 ± 12.5	23.6 ± 11.9	17.0 ± 11.2	0.029	19.9 ± 8.7	24.6 ± 11.7	23.3 ± 11.6	0.529	−5.3 ± 8.5	0.9 ± 7.0	6.3 ± 6.2	0.000
C2-C7	7.8 ± 20.2	−1.7 ± 16.2	−4.3 ± 16.2	0.137	6.2 ± 8.7	2.2 ± 14.6	2.6 ± 16.1	0.799	0.6 ± 14.5	4.1 ± 12.7	7.7 ± 13.8	0.323
C2-C7 SVA	33.7 ± 11.3	35.1 ± 19.1	29.0 ± 15.7	0.327	31.2 ± 23.0	35.5 ± 17.1	33.2 ± 15.5	0.733	−1.3 ± 15.8	1.3 ± 15.8	4.0 ± 12.1	0.551
C2-T3	7.7 ± 22.1	−4.5 ± 21.1	−9.5 ± 17.4	0.053	−0.6 ± 10.4	−6.7 ± 19.2	−3.8 ± 14.8	0.595	−8.1 ± 22.5	−1.0 ± 15.4	6.5 ± 11.7	0.019
C2-T3 SVA	54.5 ± 18.8	59.4 ± 26.2	46.9 ± 22.1	0.105	52.8 ± 28.7	63.6 ± 28.7	57.3 ± 22.5	0.470	−0.5 ± 20.4	4.7 ± 16.9	10.1 ± 14.6	0.176
C2-T1 – H	17.8 ± 23.6	8.2 ± 18.2	3.4 ± 18.6	0.113	12.2 ± 8.1	12.7 ± 17.6	12.2 ± 19.4	0.992	−3.8 ± 23.3	3.7 ± 13.6	10.1 ± 12.6	0.043
C2Slope	16.3 ± 11.6	24.7 ± 12.8	21.2 ± 10.6	0.138	14.5 ± 11.0	22.4 ± 11.9	20.6 ± 10.7	0.217	−3.8 ± 9.5	−2.4 ± 10.9	−1.1 ± 12.0	0.798
C0Slope	−1.1 ± 4.4	0.3 ± 6.9	−1.3 ± 7.5	0.762	−0.6 ± 7.4	−2.8 ± 6.9	− 2.4 ± 10.1	0.898	− 2.1 ± 7.2	−3.0 ± 7.3	−6.7 ± 10.2	0.534
C0-C2	18.3 ± 12.3	23.2 ± 8.1	22.8 ± 9.1	0.562	13.0 ± 14.5	23.1 ± 8.8	21.9 ± 7.9	0.099	−5.1 ± 3.3	−1.9 ± 7.6	− 1.9 ± 9.8	0.824
MGSlope	5.3 ± 4.5	5.2 ± 6.6	3.2 ± 6.9	0.673	4.7 ± 7.1	2.8 ± 6.4	3.2 ± 9.9	0.921	−4.3 ± 7.0	−3.1 ± 7.6	−7.2 ± 10.5	0.601

*SS* sacral slope, *PT* pelvic tilt, *PI* Pelvic incidence, *LL (L1 – S1)* lumbar lordosis measured from L1 to S1, *PI-LL* pelvic incidence minus lumbar lordosis mismatch, *TK (TK 2 – TK 12)* thoracic kyphosis measured from TK 2 to TK 12, *SVA* sagittal vertical axis, *T1SPi* T1 spinopelvic inclination, *TPA* T1 pelvic angle; cranial: *MGS* McGregor Slope (line). *C0S* C0 Slope, *C2S* C2 Slope, C2-C0 angle; C2-C7 angle, C2-C7 SVA (*SVA* sagittal vertical axis), C2-T3 angle, C2-T3 SVA (*SVA* sagittal vertical axis), T1 Slope, *MGS* McGregor Slope (line); *Δ* parameter change pre- to post-operatively; data expressed as mean ± standard deviation; *p* statistical significance, *p* < 0.05

### Stratification by change in SVA (more than 25 mm, within, and less than − 25 mm)

Baseline parameters of patients who were stratified according to their SVA revealed that patients with a post-operative increase in SVA had a smaller preoperative TK (− 31° vs. -37° vs. -44°, *p* = 0.012) and were more posteriorly aligned (T1SPi: − 7° vs. -4° vs. -1°, *p* < 0.001). Furthermore, they had a smaller pre-operative T1 Slope (15° vs. 22° vs. 27°, *p* < 0.001), larger cervical kyphosis (C2-C7: -7° vs. -2° vs. 4°, *p* = 0.05; C2-T3: -11° vs. -7° vs. 2°, *p* = 0.044) and a more posteriorly aligned cervical spine (C2-C7 SVA: 25 mm vs. 36 mm vs. 37 mm, *p* = 0.002; C2-T3 SVA: 41 mm vs. 57 mm vs. 62 mm, *p* = 0.002).

Post-operatively, these patients had a positive PI-LL mismatch (2° vs. -5° vs. -11°, *p* = 0.001), smaller LL (51° vs. 61° vs. 60°, *p* = 0.006), smaller TK (− 34° vs. -42° vs. -46°, *p* = 0.007), and were more anteriorly aligned (SVA: 20 mm vs. -5 mm vs. -20 mm, *p* = 0.003; T1SPi: − 3° vs. -5° vs. -6°, *p* = 0.022; TPA: 11° vs. 7° vs. 4°, *p* = 0.007).

AIS patients with increased SVA post-operatively had a larger ΔLL (− 5° vs. 1° vs. 3°, *p* = 0.008), larger ΔPI-LL (5° vs. -1° vs. -3°, *p* = 0.004) and anterior change in global alignment (T1Spi: 4° vs. -1° vs. -5°, *p* < 0.001; TPA: 3° vs. -1° vs. -5°, *p* < 0.001). Furthermore, ΔT1 slope, ΔC2-C7 SVA, ΔC2-T3, and ΔC2-T3 SVA significantly increased with increased SVA (ΔT1 Slope 6° vs. 4° vs. -2°, *p* < 0.001; ΔC2-C7 SVA: 8 mm vs. 0 mm vs. -2 mm, *p* = 0.034; ΔC2-T3: 7° vs. 3° vs. -4°, *p* = 0.023; ΔC2-T3 SVA: 15 mm vs. 5 mm vs. -1 mm, *p* = 0.001) (Fig. 3b) (see Table 6).

**Table 6:** Stratification by change in sagittal vertical axis (SVA): comparison of baseline and postoperative spino-pelvic parameters and their respective pre-to post-op change

	Stratification by change in SVA											
	Baseline				Post-OP				Change (Δ)			
	Decrease	Stable	Increase	p	Decrease	Stable	Increase	p	Decrease	Stable	Increase	p
SS	40.6 ± 10.4	43.7 ± 9.0	37.9 ± 11.9	0.131	40.6 ± 10.6	43.6 ± 10.4	39.1 ± 12.3	0.344	0.1 ± 6.4	−0.2 ± 5.8	1.2 ± 7.8	0.739
PT	9.5 ± 8.2	12.4 ± 9.0	14.3 ± 7.0	0.072	9.2 ± 10.2	12.0 ± 8.3	13.5 ± 8.2	0.195	−0.2 ± 6.6	− 0.5 ± 5.7	− 0.8 ± 7.1	0.940
PI	50.0 ± 12.9	56.2 ± 13.4	52.2 ± 14.5	0.287	49.9 ± 12.4	55.6 ± 12.6	52.6 ± 14.8	0.334	−0.2 ± 1.9	−0.6 ± 1.9	0.3 ± 2.0	0.198
PI-LL	−7.2 ± 17.0	−3.8 ± 9.6	−3.6 ± 10.6	0.512	−10.5 ± 13.3	−5.1 ± 11.5	1.7 ± 11.8	0.001	−3.3 ± 10.4	− 1.3 ± 8.5	5.3 ± 10.7	0.004
LL	57.3 ± 13.8	60.0 ± 9.7	55.8 ± 14.3	0.504	60.4 ± 10.9	60.7 ± 11.8	50.8 ± 15.0	0.006	3.1 ± 10.1	0.7 ± 8.2	−5.0 ± 10.9	0.008
TK	−43.5 ± 22.5	−37.2 ± 9.7	−30.5 ± 13.5	0.012	−45.6 ± 18.5	−41.6 ± 10.1	−33.8 ± 11.9	0.007	−2.0 ± 14.1	−4.4 ± 10.9	−3.4 ± 10.1	0.766
SVA	32.6 ± 44.9	1.4 ± 33.0	−26.3 ± 39.0	0.000	−19.9 ± 36.5	−5.1 ± 36.0	19.6 ± 51.4	0.003	−52.4 ± 25.8	−6.6 ± 7.8	45.9 ± 34.2	0.000
T1SPI	−1.1 ± 4.3	−4.2 ± 3.2	− 6.9 ± 3.1	0.000	−5.7 ± 4.1	− 4.7 ± 3.2	−2.8 ± 4.1	0.022	− 4.6 ± 3.5	−0.5 ± 1.5	4.0 ± 2.9	0.000
TPA	8.4 ± 7.8	8.3 ± 7.8	7.5 ± 7.1	0.897	3.6 ± 8.9	7.3 ± 7.5	10.7 ± 8.5	0.007	−4.8 ± 4.6	−0.9 ± 4.4	3.2 ± 6.2	0.000
T1Slope	26.5 ± 12.9	21.6 ± 8.9	14.6 ± 10.7	0.000	24.7 ± 14.3	25.1 ± 7.5	20.9 ± 10.6	0.311	−1.7 ± 7.9	3.5 ± 6.8	6.3 ± 6.6	0.000
C2-C7	4.0 ± 22.0	−1.7 ± 9.8	−7.0 ± 13.8	0.050	5.5 ± 17.7	4.3 ± 12.4	−0.6 ± 13.5	0.280	2.9 ± 14.1	5.9 ± 14.5	7.6 ± 12.3	0.444
C2-C7 SVA	36.7 ± 13.9	36.1 ± 14.6	25.0 ± 18.2	0.013	35.1 ± 19.0	35.9 ± 12.7	31.7 ± 17.6	0.635	−1.5 ± 13.6	−0.5 ± 14.2	7.6 ± 12.9	0.034
C2-T3	2.0 ± 27.2	−6.8 ± 10.8	−11.1 ± 14.7	0.044	−3.7 ± 22.1	−5.0 ± 12.0	−4.9 ± 13.0	0.954	−4.2 ± 17.7	2.7 ± 14.7	7.1 ± 11.2	0.023
C2-T3 SVA	62.2 ± 23.3	57.3 ± 21.9	40.9 ± 21.2	0.002	61.4 ± 32.7	63.0 ± 17.5	54.5 ± 23.4	0.445	−0.8 ± 18.3	4.8 ± 12.5	14.9 ± 13.0	0.001
C2-T1 – H	13.2 ± 26.8	7.0 ± 10.0	2.1 ± 15.3	0.117	14.5 ± 22.4	16.6 ± 14.0	7.8 ± 14.7	0.189	2.6 ± 17.6	11.0 ± 12.6	6.2 ± 13.1	0.219
C2Slope	21.6 ± 14.8	23.7 ± 9.3	21.0 ± 10.2	0.737	19.7 ± 13.6	20.7 ± 8.4	21.4 ± 11.2	0.860	−3.0 ± 10.5	−3.3 ± 13.1	0.0 ± 10.6	0.515
C0Slope	−1.4 ± 7.6	3.0 ± 6.9	−3.1 ± 5.1	0.054	−1.3 ± 6.8	−1.7 ± 4.5	−4.0 ± 13.2	0.672	−1.0 ± 7.7	−7.3 ± 8.4	−6.8 ± 9.0	0.200
C0-C2	19.1 ± 11.7	24.1 ± 7.4	24.3 ± 6.7	0.238	18.8 ± 11.3	20.8 ± 7.1	24.9 ± 7.4	0.183	−3.6 ± 8.2	−3.8 ± 9.5	1.6 ± 5.5	0.351
MGSlope	2.7 ± 6.4	9.3 ± 6.8	2.1 ± 4.2	0.011	4.3 ± 6.0	4.0 ± 5.2	1.1 ± 13.3	0.593	−0.8 ± 7.6	−8.6 ± 9.0	−8.1 ± 8.7	0.123

*SS* sacral slope, *PT* pelvic tilt, *PI* Pelvic incidence, *LL (L1 – S1)* lumbar lordosis measured from L1 to S1, *PI-LL* pelvic incidence minus lumbar lordosis mismatch, *TK (TK 2 – TK 12)* thoracic kyphosis measured from TK 2 to TK 12, *SVA* sagittal vertical axis, *T1SPi* T1 spinopelvic inclination, *TPA* T1 pelvic angle; cranial: *MGS* McGregor Slope (line). *C0S* C0 Slope, *C2S* C2 Slope, C2-C0 angle; C2-C7 angle, C2-C7 SVA (*SVA* sagittal vertical axis), C2-T3angle, C2-T3 SVA (*SVA* sagittal vertical axis), T1 Slope, *MGS* McGregor Slope (line); *Δ* parameter change pre- to post-operatively; data expressed as mean ± standard deviation; *p* statistical significance, *p* < 0.05

**Fig. 4:**
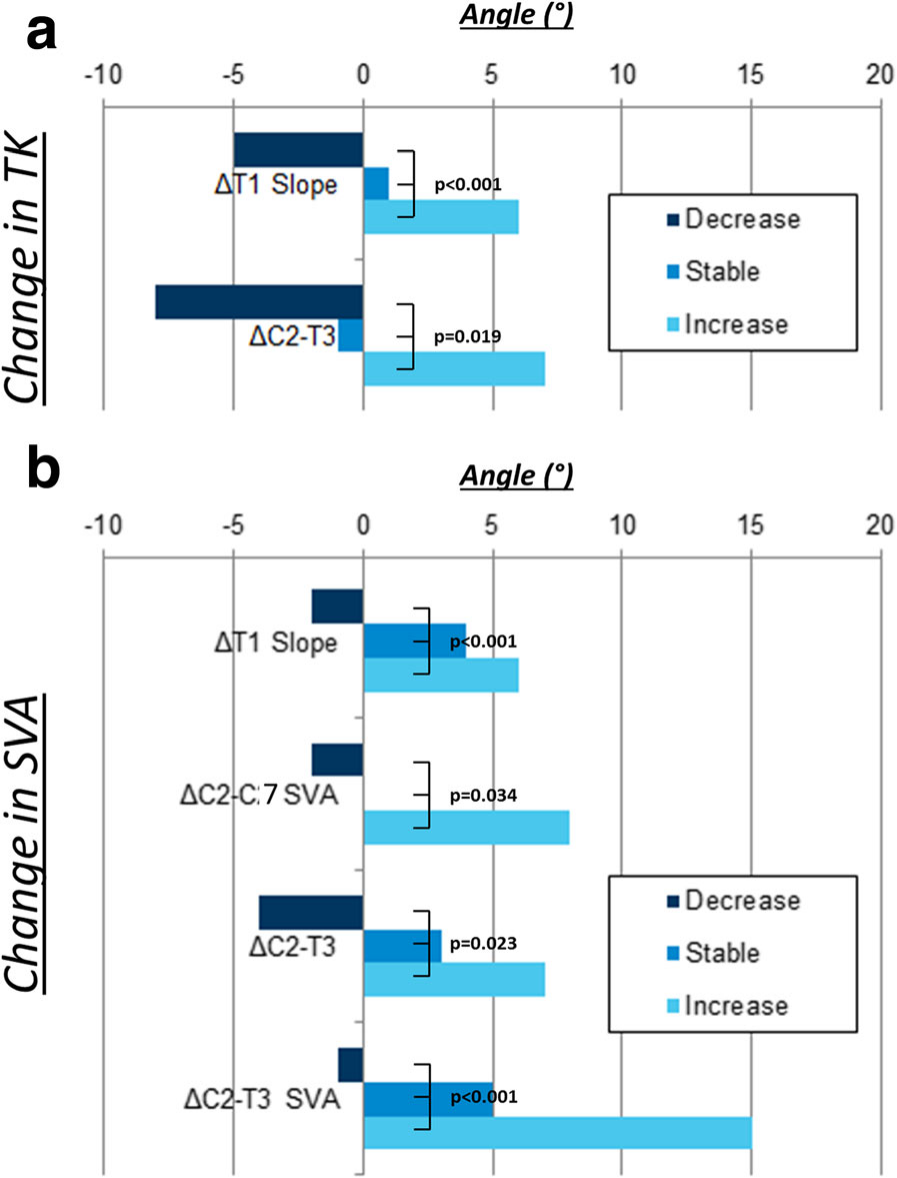
**a** Diagram with correlation analysis of subgroup: pre-to post-operative change in thoracic kyphosis (TK) with ΔT1 Slope, ΔC2-C7, ΔC2-T3, ΔC2-T3 SVA; *p* = statistical significance, *p* < 0.05. **b** Diagram with correlation analysis of subgroup: pre-to post-operative change in sagittal vertical axis (SVA) with ΔT1 Slope, ΔC2-T3, ΔC2-T3 SVA; *p* = statistical significance, *p* < 0.05

Regardless of patient stratification (TK or SVA), no statistical change in upper cervical parameters (C2 Slope, C0 Slope, C0-C2) was noted. Finally, the correlation analysis showed that with a post-operative increase in ΔTK, cervical spine curvature became more lordotic. ΔTK significantly correlated with ΔT1 Slope (*r* = 0.630; *p* < 0.001), ΔC2-C7 (*r* = 0.274; *p* = 0.021), ΔC2-T3 (*r* = 0.428; *p* < 0.001), ΔC2-T3 SVA (*r* = 0.308; *p* = 0.009) (Fig. 4a). Similarly, cervicothoracic spine curvature became more lordotic when ΔSVA increased post-operatively. There was a significant correlation between ΔSVA and ΔC2-T3 (*r* = 0.398; *p* = 0.001) and ΔC2-T3 SVA (*r* = 0.280; *p* = 0.018) (Fig. 4b).

## Discussion

A long-held belief in the field of cervical spine research was that a kyphotic cervical spine was a source of pathology [29, 30]. Lordosis was considered the only normal alignment [1]. The association of cervical kyphosis with a higher likelihood of concomitant diseases like cervical myelopathy or accompanying symptoms such as neck pain may have been a major contributor to the vilification of kyphotic alignment [29].

Le Huec et al. observed that almost 30% of their asymptomatic young volunteers had a kyphotic cervical alignment [3].This finding, the presence of cervical kyphosis in young patients with no signs of pathology and no complaints, has begun to trigger a paradigm shift in conceptualizing the physiologic alignment of the cervical spine [2, 5, 22]. This has motivated many investigators to dig deeper into the complex realm of cervical alignment.

Cervical alignment is not an independent entity and should be viewed through a wider lens; global and regional alignments play hidden roles and are key players in determining cervical curvature [9, 18]. In the context of AIS, complex 3D deformation of normal regional alignment (TK) can change cervical alignment [19–21, 31]. Understanding the intricate relationship between sagittal alignment and the cervical spine is essential for predicting its effects on cervical curvature.

In conservatively treated AIS patients, it has already been shown that the aforementioned relationship exists [32]. In this study, we hypothesized that operative treatment of AIS patients with rod-screw contouring would influence cervical alignment by altering the sagittal profile.

Our data shows that patients with pre-operative kyphosis in the lower cervical spine had a low TK and low T1 slope, which is in line with former studies that revealed T1 Slope to have an influence on cervical curvature [19–21, 31, 33, 34]. Specifically, when T1 slope decreases, the cervical spine becomes more kyphotic. In accordance with increases in T1 slope, their lower cervical spines (cervicothoracic: C2-T3) became more lordotic after surgery. The spine of AIS patients is flexible and therefore malleable through corrective surgery. When thoracic kyphosis is accentuated, the lumbar spine compensates with increasing lordosis to maintain spinal balance and position within the cone of economy [35]. Increased TK results in increased T1 Slope [33], and an increased T1 Slope has a direct effect on cervical curvature resulting in more cervical lordosis [33] as a form of compensation. In other words, the increase in lower cervical lordosis (C2-C7, C2-T3) that we observed was a compensatory mechanism to position the head near the center of gravity (C2-C7, C2-T3).

Patient stratification based on post-operative SVA unveiled important observations in terms of preoperative global, regional and cervical parameters. It also revealed how ΔSVA correlated with the aforementioned parameters. We noted that patients who exhibited an increase in SVA after surgery had a lower preoperative SVA, TK, and T1 Slope and were more posteriorly aligned (T1SPi) when compared to the subgroup of patients who had a decreased post-op SVA or a stable SVA. They also had a kyphotic lower cervical spine before surgery. This cervical kyphosis could also be explained by compensation attempt to position the head near the center of gravity [36]. Notably, in this subgroup of patients, TK significantly decreased without affecting T1 Slope or cervical curvature, which could be explained by the increased anterior alignment (SVA) post-operatively.

When we studied the effect of pre- to post-operative change (Δ), we noted that in the subgroup with postoperative increases in SVA, there was also a significant increase of ΔT1 Slope, ΔC2-C7 SVA, and ΔC2-T3 SVA. Thus, post-operative anterior shift in global alignment is associated with a post-operative anterior change in cervical and cervicothoracic alignment. Interestingly, ΔC2-C7 did not significantly change; on the other hand, ΔC2-T3 revealed a significant increase, denoting more lordosis of the cervicothoracic junction. This implies that changing SVA after surgery affects cervical sagittal alignment and changes the cervicothoracic junction with no significant effect on local cervical curvature (C2-C7).These findings are in line with findings from Diebo et al. In their adult population, the highest cSVA was observed in the subgroup whose SVA was > 50 mm [37].

TK and SVA correlated with lower cervical spine parameters. Specifically, we found that TK correlates with LL, T1 Slope, C2-C7, C2-T3, and C2-T3 SVA. Furthermore, SVA correlates with LL, C2-T3, and C2-T3 SVA. This affirms a few concepts: first, there is interdependence between regional spine parameters (TK), global alignment (SVA), and lower cervical curvature. Second, in AIS patients treated with fusion surgery, there is a range of motion reserve in unfused segments that can be recruited for compensation. Yang et al. previously discussed how unfused segments might play an important compensatory role in maintaining sagittal balance [35]. Finally, we took a step back from the lower region of the cervical spine and examined upper cervical spine parameters. No significant difference was found in terms of pre- to postoperative analysis of the influence of global and regional alignment on upper cervical alignment in AIS patients. We suggest two possible explanations. First, the lower cervical spine alone is able to compensate over five segments of motion to position the head sufficiently close to the center of gravity, making it unnecessary to recruit the upper cervical spine. Second, the upper cervical spine may have been recruited to achieve normal horizontal gaze.

In the past few years, the interdependence between TK, T1Slope, and SVA and their impact on the cervical spine in the context of degenerative disease has been proven [38]. In our study, we have shown in detail that this relationship also exists in AIS patients.

Incidence of prolonged neck pain after spinal surgery has been well described in previous studies, in which it was evident from the lower scores of patient-reported health-related quality of life (HRQOL) parameters [13, 39]. This makes it important to undertake further investigations to develop an understanding of the patho-mechanism of this post-operative cervical pain, with the goal of devising therapeutic or preventative strategies.

Comprehensive preoperative planning may be a potential point of intervention. Whether prevention of post-operative cervical kyphosis by thoracic or thoraco-lumbar fusion in AIS patients leads to better clinical outcomes or does not merits a prospective investigation. Furthermore, it is already known that the presence of cervical kyphosis during a degenerative process can lead to intervertebral disc degeneration, spondylotic myelopathy, and elongation of the spinal cord [40]. None of our patients exhibited neck pain post-operatively at time of conduction of this study. However, due to the retrospective nature of our study, we could not follow up with our patients to investigate whether they developed neck pain. This constitutes one of the limitations of our study. Hence, prospective investigations are needed to assess whether post-operative cervical kyphosis in AIS patient would represent the beginning of a pathological process that would end in cervical pain.

A further limitation of our study is a potential selection bias which would jeopardize the external validity of our results as no randomization was utilized while choosing the full-spine radiographs. Furthermore, we concede that we could not stratify our study population based on Lenke classification due to resulting very small subgroups. Thus, we considered the pre- to post-operative TK and SVA to be more appropriate for our study cohort. These limitations notwithstanding, our retrospective pre- to post- analysis revealed important correlations and enabled us to disentangle complex relationships between different parts of the spine both before and after surgery.

## Conclusion

Cervical curvature is influenced by post-operative changes in TK, T1 Slope, and SVA. Interestingly, all variations of the cervical parameters pre- to post-operatively were restricted to the lower cervical spine with no effect on the upper cervical spine, regardless of how patients were stratified. Due to the post-operative impact of AIS surgery on cervical alignment, we suggest that lower cervical alignment should be considered in preoperative planning.

## Abbreviations

C0S: C0 Slope
C2-C7 SVA: (SVA = sagittal vertical axis)
C2S: C2 Slope
C2-T3 SVA: (SVA = sagittal vertical axis)
DICOM: Digital Imaging and Communications in Medicine
HRQOL: health-related quality of life
LL (L1 – S1): lumbar lordosis measured from L1 to S1
MGS: McGregor Slope (line)
PACS: Picture archiving and communication system
PI: Pelvic incidence
PI-LL: pelvic incidence minus lumbar lordosis mismatch
PT: pelvic tilt
SS: sacral slope
SVA: sagittal vertical axis
T1SPi: T1 spinopelvic inclination
TK (TK 2 – TK 12): thoracic kyphosis measured from TK 2 to TK 12
TPA: T1 pelvic angle
